# Cardiovascular disease treatment and hyperkalemia: A report of three cases

**DOI:** 10.3892/mi.2025.253

**Published:** 2025-07-18

**Authors:** Saime Paydaş, Aydin Halefoğlu

**Affiliations:** 1 Department of Internal Medicine Nephrology, Acibadem Adana Hospital, 01130 Seyhan/Adana, Turkey; 2 Cardiovascular Intensive Care Unit, Acibadem Adana Hospital, 01130 Seyhan/Adana, Turkey

**Keywords:** cardiovascular disease, renin angiotensin system inhibitors, hyperkalemia, potassium binders

## Abstract

Renin-angiotensin-aldosterone system inhibitors (RAASi) are primarily used for the treatment of hypertension and diabetic/non-diabetic reno-cardiovascular diseases. The present study describes the cases of 3 patients with hyperkalemia that occurred during RAASi therapy and describes the therapeutic approach used for this serious complication. The clinical/laboratory findings of hospitalized patients with hyperkalemia within a short time period (within 30 days) and treatment were recorded. Acute hyperkalemia developed due to angiotensin converting enzyme inhibitor (ACEi)/angiotensin receptor blocker (ARB) and spironolactone in 3 patients, whose cases are reported herein. Insulin glucose infusion (IGI) + salbutamol + potassium binders were used for the treatment of severe hyperkalemia. Acute kidney injury (AKI) regressed in all patients. Hyperkalemia was corrected within 6 h in 1 patient who was treated with sodium zirconium cyclosilicate in addition to IGI. On the whole, the present study demonstrates that hyperkalemia may be symptomatic/asymptomatic and may develop during the earlier or later period of RAASi therapy for cardiovascular-renal disease, particularly in older patients. AKI improved with the correction of hyperkalemia and the discontinuation of ACEi/ARB and spironolactone. Sodium zirconium cyclosilicate may be the preferred treatment in emergency cases of hyperkalemia due to its rapid effects. On the other hand, sodium glucose co-transporter 2 inhibitors and non-steroidal mineralocorticoid receptor antagonists may also be used to avoid the development of hyperkalemia in patients undergoing RAASi therapy.

## Introduction

Hyperkalemia is a critical electrolyte disorder frequently encountered in emergency departments. Hyperkalemia can affect muscle contraction and myocardial excitability and can cause fatal arrhythmia/sudden death and requires rapid treatment. Patients with diabetes, heart failure and/or renal failure treated with renin-angiotensin-aldosterone system inhibitors (RAASi) are at an increased risk of developing hyperkalemia (1,2). On the other hand, both hyperkalemia and the discontinuation of RAASi are associated with an increased risk of cardiovascular events, hospitalizations and mortality. RAASi are recommended drugs used in the treatment of hypertension and cardiovascular/renal diseases. They are preferred due to their protective effects on cardiovascular diseases, diabetic nephropathy and a number of kidney diseases (1-3).

However, RAASi therapy can cause acute and chronic hyperkalemia and this is a critical adverse effect in daily practice. Acute hyperkalemia should be treated immediately by stabilizing the cardiac membrane requires followed by shifting potassium into cells, to decrease the total body potassium content in the kidneys and gastrointestinal route or hemodialysis. Although chronic hyperkalemia develops following a longer period of RAASi therapy and the manifestations of the condition tend to be less severe, it is not benign and has been associated with increased morbidity and mortality rates (1,4). In clinical practice, dietary potassium restriction, diuretic therapy and the correction of acidosis, if present, are commonly performed for the treatment of hyperkalemia. Dose reduction and/or the discontinuation of RAASi therapy is not advisable, as these drugs have positive effects in patients with heart failure and proteinuric kidney disease (2,3). RAASi therapy is considered the optimal therapy for patients with cardiovascular-renal disease. A potassium-restricted diet, particularly new potassium binders [such as sodium zirconium cyclosilicate (SZC) and patiromer] are necessary to prevent hyperkalemia associated with RAASi. Other alternatives are sodium-glucose cotransporter 2 inhibitors; these drugs may be helpful in maintaining the use of RAASi. Additionally, RAASi cause kidney injury and this effect limits the use of RAASi in some cases. Recently, it was shown that non-steroidal mineralocorticoids cause less hyperkalemia and are recommended for cardiovascular disease, diabetic kidney disease for their cardio-renoprotective effects (1).

The present study describes the cases of 3 patients with severe hyperkalemia which developed due to RAASi and describes the treatment of this emergent side-effect.

## Case report

The present study describes the cases of 3 patients hyperkalemia requiring hospitalization, due to RAASi within 1 month. The detailed clinical and laboratory findings and drugs used for all 3 patients are presented below:

### Case 1

A 72-year-old liver transplant recipient was hospitalized in Adana Acibadem Hospital due to severe hyperkalemia and acute kidney injury (AKI). He had a history of diabetes mellitus (DM), hypertension, coronary artery disease and heart failure with a reduced ejection fraction (HFrEF). Cadaveric liver transplantation was performed 14 months prior. The patient was admitted to the emergency room due to fatigue at 5 days following angiotensin converting enzyme inhibitor (ACEi) and spironolactone treatment. His serum potassium level was 6.5 mEq/l and he was hospitalized due to drug-related hyperkalemia and AKI. The blood pressure, pulse and body temperature of the patient were within normal limits. No ECG changes or metabolic acidosis were detected. The treatment of hyperkalemia, which included calcium gluconate 10 ml of a 10% solution administered intravenously, insulin glucose infusion (IGI; short acting insulin at 10 IU in 250 ml %10 glucose, 50 ml/h ventolin inhaler (10-20 mg dose in 4 ml saline delivered by a nebulizer) and sodium polystyrene sulfonate (3x15 g/day, was commenced. When IGI therapy was terminated, the serum potassium was level was 6.3 mEq/l at the 32nd hour of hospitalization. Following the addition of SZC (2x5 g/day), the potassium level decreased from 6.3 mEq/l to 5.5 mEq/l within 6 h. During follow-up, the serum potassium level was 4.32 and the serum creatinine level was 1.23 mg/dl. The demographic characteristics of the patient are summarized in Table I.

### Case 2

A 58-year-old morbidly obese diabetic female patient was being treated with insulin, gliclazide, empagliflozin, ramipril and spironolactone in Adana Acibadem Hospital). The serum potassium level was 6.75 during the cardiology check-up. Metabolic acidosis and ECG changes were not detected. She was hospitalized and treated with IGI (short acting insulin 10 IU in 250 ml %10 glucose, 50 ml/h, ventolin inhaler (10-20 mg dose in 4 ml saline delivered by a nebulizer) and sodium polystyrene sulfonate (3x15 g/day). Following 36 h of IGI, the serum potassium was level was ~5.18 mEq/l and 1 week later, her serum potassium level was 4.5 mEq/l (Table I).

### Case 3

A 94-year-old female patient was being treated with digoxin, spironolactone and candesartan due to hypertension and ischemic heart disease in the First Line Center Adana Seyhan Family Medicine Polyclinic. Hyperkalemia (serum potassium level, 6.6 mEq/l) was detected during routine blood tests in primary care control. The blood pressure and pulse rates of the patient were normal. There was a previously known left bundle branch block on the ECG and no new changes developed. Acidosis was not detected. Hyperkalemia was corrected with 6 h with IGI treatment in the emergency room of Adana Seyhan Hospital, Adana, Turkey. She was then discharged. A potassium-restricted diet and sodium polystyrene sulfonate were recommended. The patient was then followed-up at home Spironolactone and candesartan were discontinued. In the follow-up period, hyperkalemia did not develop and the serum creatinine level decreased from 1.97 to 1.33 mg/dl (Table I).

In all patients with hyperkalemia, RAASi was discontinued and calcium gluconate was administered intravenously in all patients. IGI and salbutamol inhaler were commenced. Intravenously furosemide was administered in a patient with hypervolemia (Case 3). The serum potassium level was measured at frequent intervals. Additionally, treatment with oral potassium binders, namely sodium polystyrene sulfonate was commenced. Despite the interruption of IGI, patients with a serum potassium level of 5.5 mEq/l were discharged.

## Discussion

The combination of ACEi/angiotensin receptor blocker (ARB) and mineralocorticoid receptor antagonists (MRAs) is recommended as the first step in patients with cardiovascular disease and HFrEF (2). However, in these patients, concomitant DM and renal failure increase the risk of developing hyperkalemia. The prevalence of hyperkalemia has been reported as 2.9% in hospitalized patients and 11.5% in patients with heart failure (2,3).

The development of life-threatening severe hyperkalemia is an issue which limits the use of RAASi in patients with cardiovascular disease and DM. The present study describes the cases of 3 patients with hyperkalemia. These 3 patients were using spironolactone + ACEi/ARB combination. In case 1, Acute kidney disease and/or renal tubular acidosis type 4 may develop secondary to nephrotoxicity associated with CNI (4). However, hyperkalemia did not worsen or persist in this diabetic patient despite continuing CNIs. A combination of MRAs + RAASi was used in the 3 patients described herein. The cardio-renoprotective effect of dual RAASi in diabetic patients, has been demonstrated in a number of studies. In a previous meta-analysis, depending on the discontinuation of RAASi, it was shown that the risk of mortality due to all causes [hazard ratio (HR), 1.41], end-stage renal disease (HR 1.23) and major adverse cardiac events (MACE) (HR, 1.20) increased. A decrease in the risk of hyperkalemia (HR, 0.79) was detected (5). The authors of that study suggested that RAASi therapy could reduce the progression of chronic kidney disease (5). The use of RAASi or other disease-modifying drugs was previously evaluated in 238 patients with HFrEF (6). According that study, ACEI/ARB/angiotensin receptor-neprilysin inhibitor + β-blocker + MRA and sodium glucose co-transporter 2 inhibitor (SGLT2i) therapy should be used for the treatment of HFrEF (6). Of note, ~90% of dietary potassium is excreted through the kidneys. Potassium is balanced in the kidney; it is regulated passively by secretion in the distal convulated tubules and cortical collecting ducts, particularly in principal cells. Luminal flow in the distal nephron, luminal sodium concentration and epithelial sodium channel activity in response to aldosterone may cause hypokalemia or hyperkalemia. In patients with hyporeninemic hypoaldosteronism, the risk of developing hyperkalemia increases due to the decrease in renin release due to damage to the juxtaglomerular apparatus and decrease in aldosterone release in the adrenal gland (7-9). In patients with diabetic kidney disease, the frequency of hyperkalemia in patients taking ARB/ACEI using single RAASi has been reported as follows: ACEI [odds ratio (OR), 3.07; 95% CI, 1.14 to 8.31)], ARB (OR, 2.57) (10).

Although not observed due hyperkalemia, BRASH SYNDROME with the five findings including bradycardia, renal failure, atrioventricular nodal block, shock and hyperkalemia can develop (11). Patients with BRASH syndrome should be treated with atropine and noradrenaline in addition to treatment for hyperkalemia. It is known that noradrenaline infusion facilitates the entry of potassium into cells. In a previous study, the annual prevalence of hyperkalemia was found to be 4.41%; the risk factors were an age >65 years, decreased GFR, DM, use of RAAS blockers and patients on hemodialysis (12). SGLT2i are known to have positive effects on mortality in patients with chronic kidney disease and heart failure (10). Furthermore, the addition of SGLT2i to different MRA + ACEI/ARB combinations, reduces the development of hyperkalemia (10).

Considering the cardiovascular protection of RAAS inhibitors, SZC can be added to the treatment for hyperkalemia; SZC has been suggested to be cost-effective (13). By contrast, the REALIZE-K study (Study to assess efficacy and safety of SZC for the Management of High Potassium in Patients with Symptomatic HFrEF Receiving Spironolactone) compared SZC with a placebo for efficacy and safety in patients with heart failure reduced EF and hyperkalemia during optimal dose MRA treatment (14). It was found that the number events of edema and hospitalizations for heart failure were greater in the group using SZC than the placebo; however, the number of heart failure events was minimal. However, gastrointestinal sodium absorption was not evaluated in the present case report; thus, this may be a limitation of the present study. Perhaps new data are required on SZC and sodium retention (14).

Notably, in the present study, in case 1, hyperkalemia resolved with SZC after 6 h, although IGI was discontinued. Sodium polystyrene sulfonate (SPS) can also be used a gastrointestinal potassium binders. However, there are no clear data available on whether SPS or SZC should be used as a gastrointestinal potassium binder for the treatment of hyperkalemia. In a previous study, it was found that SZC and SPS had similar effects on the rate of normokalemia at 48 h, although the incidence of hypertension and side-effects were less common in patients who received SZC (15). However, the earlier onset of the effect may be beneficial in the treatment of emergent hyperkalemia.

Of note, herein, cases 2 and 3, who used the the ACEi + aldactone combination for a longer time period, were asymptomatic and hyperkalemia was determined in routine control tests. Due to the development of hyperkalemia, ARB and MRA were discontinued in these patients. However, in a previous study, in an evaluation involving 248,963 patients, the discontinuation of RAAS inhibitors was found to increase all-cause mortality (HR, 1.41), end-stage renal disease (HR, 1.32) and MACE (HR, 1.20), while decreasing the risk of hyperkalemia (0.79) (5). The authors of that study suggested that the continuation of RAAS blockers is beneficial in patients with chronic kidney disease (5).

As demonstrated herein, within a short period of 1 month, hyperkalemia developed in 3 cases who were followed-up for cardiovascular disease. Hyperkalemia and acute kidney disease may develop symptomatically or incidentally in patients administered RAASi. Therefore, more frequent monitoring or anti-potassium treatment may be initiated in patients with DM, those aged >65 years,, those with chronic kidney disease, those on dialysis, and those using ACEI/ARB (12). The restriction of dietary potassium may not be possible for a healthy diet. Since patients with hyperkalemia may be asymptomatic, it is difficult to determine how often serum potassium levels should be examined.

It is known that the concomitant use of SGLT2 inhibitors in patients with HFrEF may reduce the risk of hyperkalemia (10). The risk of hyperkalemia when adding SGLT2 inhibitors to ACEI/ARB or MRA or ACE/ARB+MRA treatment regimens has been found to be less frequent. In addition, non-steroidal MRAs cause less frequent hyperkalemia than steroidal MRAs (10). SZC can be added to RAASi if there is a risk of hyperkalemia. In a previous study, SZC was suggested to be cost-effective among patients treated with RAASi (13). Sodium polystyrene sulfonate can also be used as a gastrointestinal potassium binder. However, there are no clear data on whether sodium polystyrene or sodium zirconium should be used as a gastrointestinal potassium binder for hyperkalemia treatment. However, the earlier-onset effect of SZC may be beneficial in the treatment of emergent hyperkalemia.

In conclusion, the present study demonstrates that during treatment with RAASi in clinical practice, symptomatic/asymptomatic hyperkalemia and AKI may develop in the early/later periods during therapy for cardiovascular-renal disease, particularly in older patients. It would be pertinent to follow-up patients more frequently for hyperkalemia, and patients should be educated about potassium-rich foods. In addition, the present study wished to emphasize that selecting non-steroidal MRAs and adding SGLT2i to the treatment regimen is safer for patients and clinicians. Proving the accuracy of using oral potassium binders such as SZC, the effects of which can begin at an early stage, in emergency treatment, may provide significant convenience in treatment.

## Figures and Tables

**Table I:** Demographic characteristics of the patients in the present case report.

Characteristic	Case 1	Case 2	Case 3
Age, years/sex	72/M	58/F	94/F
Co-morbid condition	DM + rEFHF + IHD + Liver Tx	DM + pEFHF + IHD + BMI >40	pEFHF + IHD + TIA 140/85
SBP/pulse/min	110/70	130/85	
Spironolactone/ACEI/ARB	+/+	+/+	+/+
Other drugs	a, b, c, d, e, f, g, i, k	a, b, c, d, e, f, i, k	a, e, f, i, l
Date of presentation, name of hospital	January 10, 2024 AAH	January 24, 2024 AAH	February 2, 2024 ASGH
Serum K 1 mEq/dl	6.5	6.75	6.6
Serum K 2 mEq/dl	5.39	5.18	5.3
Serum K 3 mEq/dl	4.32 (July 22, 2024)	4.5 (June 8, 2024)	4.4 (June 5, 2024)
Creatinine 1 mg/dl	1.57	2.61	1.97
Creatinine 2 mg/dl	1.17	2.40	1,33
Creatinine 3 mg/dl	1.23 (July 22, 2024)	1.83 (June 8, 2024)	1.44 (June 5, 2024)
IGI duration	36 h	36 h	6 h

M, male; F, female; DM, diabetes mellitus; K, potassium; pEFHF, heart failure with preserved ejection fraction; rEFHF, heart failure with reduced ejection fraction; IHD, ischemic heart disease; IGI, insulin glucose infusion; SBP, systolic blood pressure (mmHg); Tx, transplantation; TIA, transient ischemic attack; AAH, Adana Acıbadem Hospital; GH, Adana Seyhan Government Hospital. As regards serum K levels and creatinine levels, the numbers indicate the following: 1, baseline level; 2, control level; 3, last visit. For ‘other drugs’, these are indicated as follows: a, furosemide; b, calcium channel blocker; c, proton pump inhibitor; d, insulin; e, dipyridamole; f, aspirin; g, calcineurin inhibitor; h, mycophenolate mofetil; I, β-blocker; k, lipid lowering drug (atorvastatin in case 1 and case 2 or rosuvastatin in case 3); l, digoxin.

## Data Availability

The data generated in the present study may be requested from the corresponding author.
